# Racial and Ethnic Disparities in Take-Home Methadone Use for Medicare Beneficiaries With Opioid Use Disorder

**DOI:** 10.1001/jamanetworkopen.2024.31620

**Published:** 2024-08-30

**Authors:** Sugy Choi, Yutong Zhang, Mark Aaron Unruh, Emma E. McGinty, Hye-Young Jung

**Affiliations:** 1 Department of Population Health, New York University Grossman School of Medicine, New York, New York; 2 Department of Population Health Sciences, Weill Cornell Medical College, New York, New York

## Abstract

This cross-sectional study examines racial and ethnic disparities in take-home methadone use among Medicare beneficiaries with opioid use disorder following the Substance Abuse and Mental Health Services Administration’s policy change in March 2020.

## Introduction

Opioid overdose deaths in the US increased from 21 089 in 2010 to 81 806 in 2022.^1^ In response to this public health crisis, Medicare initiated coverage of methadone treatment for opioid use disorder (OUD) in January 2020, including take-home dosing for beneficiaries in opioid treatment programs (OTPs).^2^ In March 2020, in response to the outbreak of the COVID-19 pandemic, the Substance Abuse and Mental Health Services Administration (SAMHSA) increased flexibility regarding take-home methadone, which has been associated with increased treatment engagement and improved patient satisfaction with minimal medication misuse.^3^

Previous research^4,5^ indicates that Black and Hispanic patients are less likely to receive medication for OUD than White patients. It is not known whether similar disparities in take-home methadone use existed among Medicare beneficiaries following the program’s coverage of methadone and SAMHSA’s expansion of treatment flexibilities for OUD. We examined racial and ethnic disparities in take-home methadone use among beneficiaries with OUD following the implementation of these policies.

## Methods

This cross-sectional study was approved by the Weill Cornell institutional review board. The STROBE reporting guideline was followed. Informed consent was not needed because of minimal risk and the inability to conduct the study without it being waived, in accordance with 45 CFR §46. We analyzed claims for a 20% national sample of Medicare fee-for-service beneficiaries from April through December 2020 using the Medicare Beneficiary Summary File (MBSF) for data on demographics, reason for Medicare entitlement, and chronic conditions. Methadone treatment for OUD was identified in the Outpatient and Carrier Files using *Current Procedural Terminology* codes G2078 (OTP treatment) and G2067 (take-home). Rurality of a beneficiary’s residence was derived from the Area Health Resources File. Our sample included beneficiaries with at least 1 record of methadone treatment for OUD. Our outcome was a binary indicator of whether a beneficiary had at least 1 take-home methadone treatment in a month where the individual received any type of methadone treatment.

Beneficiary characteristics included race and ethnicity measures for Hispanic, non-Hispanic Black, and non-Hispanic White individuals from the MBSF, which are generally collected from the Social Security Administration. Other categories were excluded because of measurement error concerns with Medicare data.^6^ Additional characteristics included sex, dual-eligibility, reason for Medicare entitlement, rural location, number of chronic conditions, and indicators for the 10 most common chronic conditions, because more complex patients may not be appropriate candidates for take-home methadone. Statistical methods are shown in the eAppendix in Supplement 1.

## Results

The study sample included 9172 unique beneficiaries (median [IQR] age, 57 [46-65] years; mean [SD] age, 55.2 [11.9] years; 3780 [41.2%] female; 5392 [58.8%] male; 59 076 beneficiary-months) who received care in 950 OTPs. Among them, 1371 (14.9%) were Black, 915 (10.0%) were Hispanic, and 6886 (75.1%) were White. Overall, 41.2% of beneficiaries (3780 individuals) received take-home methadone, including 38.5% of Black beneficiaries (528 individuals), 43.1% of Hispanic beneficiaries (394 individuals), and 41.5% of White beneficiaries (2858 individuals).

In adjusted analysis, Black beneficiaries had an 8.4 percentage point (95% CI, −10.7 to −6.1 percentage points) lower likelihood of take-home methadone use vs White beneficiaries (Table). Using the proportion of all beneficiaries who received any take-home methadone treatment as the denominator (41.2%), this translates to a 20.4% relative difference. The adjusted difference between Hispanic and White beneficiaries was not statistically significant (−1.9 percentage points; 95% CI, −4.2 to 0.5 percentage points).

**Table.** Adjusted Estimates of Take-Home Methadone Use by Race and Ethnicity Among Medicare Beneficiaries Using Methadone to Treat Opioid Use Disorder, April Through December 2020^a^

Race and ethnicity	Estimated take-home methadone use, % (95% CI)	Difference, percentage points (95% CI)^b^
Hispanic	38.5 (35.6 to 41.3)	−1.9 (−4.2 to 0.5)
Non-Hispanic Black	31.9 (29.2 to 34.7)	−8.4 (−10.7 to −6.1)
Non-Hispanic White	40.3 (38.3 to 42.3)	0 [Reference]

^a^ The analysis was based on a sample of 9172 unique Medicare fee-for-service beneficiaries (59 076 beneficiary-months) in 2020. Among these beneficiaries, 7114 (77.6%) were dually eligible; 1348 (14.7%) were entitled to Medicare for being aged 65 years or older, 7448 (84.5%) for disability, 51 (0.6%) for end-stage kidney disease, and 25 (0.3%) for both disability and end-stage kidney disease; and 430 (4.7%) resided in rural locations. The mean (SD) number of comorbid chronic conditions among beneficiaries in the sample was 10.4 (4.9). The prevalence of the 10 most common chronic conditions was 53.4% (n = 4897) for tobacco use disorders, 45.7% (n = 4190) for anxiety disorders, 40.5% (n = 3718) for hypertension, 39.0% (n = 3580) for chronic pain fatigue and fibromyalgia, 25.9% (n = 2375) for rheumatoid arthritis and/or osteoarthritis, 25.3% (n = 2322) for viral hepatitis (general), 22.3% (n = 2043) for obesity, 22.2% (n = 2039) for chronic kidney disease, 21.7% (n = 1989) for hyperlipidemia, and 20.9% (n = 1917) for diabetes.

^b^ For details of the statistical methods, see the eAppendix in Supplement 1.

## Discussion

In this nationwide cross-sectional study, Black beneficiaries were less likely to use take-home methadone vs White beneficiaries following Medicare reimbursement for methadone and increased treatment flexibilities by SAMHSA during the initial 9 months of the COVID-19 pandemic. Potential factors associated with this disparity, including availability of take-home dosing, clinician and patient dynamics, patient preferences, and racial and ethnic bias, warrant future study. A limitation was our inability to identify candidates deemed inappropriate for take-home methadone use by OTPs. Our findings highlight the imperative to reduce inequities in OUD treatment as part of measures to effectively address the nation’s opioid public health emergency.

## Notes

Supplement 1. **eAppendix.** Statistical Methods

Supplement 2.
Data Sharing Statement
