# Supplementary material for: Racial and Ethnic Disparities in Take-Home Methadone Use for Medicare Beneficiaries With Opioid Use Disorder
Source: JAMA Netw Open. 2024 Aug 30;7(8):e2431620. doi: 10.1001/jamanetworkopen.2024.31620 (PMC11364990; doi:10.1001/jamanetworkopen.2024.31620)
Supplement: Supplement 1. — eAppendix. Statistical Methods [file jamanetwopen-e2431620-s001.pdf]

Supplemental Online Content

Choi S, Zhang Y, Unruh MA, McGinty EE, Jung HY. Racial and ethnic disparities in take-home methadone use for Medicare beneficiaries with opioid use disorder. *JAMA Netw Open*. 2024;7(8):e2431620. doi:10.1001/jamanetworkopen.2024.31620

eAppendix. Statistical Methods

This supplemental material has been provided by the authors to give readers additional information about their work.

© 2024 Choi S et al. *JAMA Network Open.*

eAppendix. Statistical Methods

A multilevel mixed-effects maximum likelihood regression model was used to estimate take-home methadone use across racial and ethnic groups in percentage points, adjusting for calendar month, age, sex, dual-eligibility, reason for Medicare entitlement, number of chronic conditions, the 10 most common chronic conditions in the study population (ie, tobacco use disorders, anxiety disorders, hypertension, chronic pain fatigue and fibromyalgia, rheumatoid arthritis and/or osteoarthritis, viral hepatitis [general], obesity, chronic kidney disease, hyperlipidemia, and diabetes), and rural residence. Indicators for opioid treatment programs (OTPs) were also included, which yielded estimates based on comparisons of beneficiaries within the same OTPs. The unit of analysis was the beneficiary-month, and SEs were adjusted for clustering at the OTP level. Analyses were performed using Stata statistical software version 17.0 (StataCorp).

© 2024 Choi S et al. *JAMA Network Open.*
