# Supplementary material for: Racial and Ethnic Disparities in Take-Home Methadone Use for Medicare Beneficiaries With Opioid Use Disorder
Source: JAMA Netw Open. 2024 Aug 30;7(8):e2431620. doi: 10.1001/jamanetworkopen.2024.31620 (PMC11364990; doi:10.1001/jamanetworkopen.2024.31620)
Supplement: Supplement 2. — Data Sharing Statement [file jamanetwopen-e2431620-s002.pdf]

# Data Sharing Statement

Choi. Racial and Ethnic Disparities in Take-Home Methadone Use for Medicare Beneficiaries With Opioid Use Disorder. *JAMA Netw Open*. Published August 30, 2024. doi:10.1001/jamanetworkopen.2024.31620

**Data**
**Data available:** No

**Additional Information**
**Explanation for why data not available:** Our data use agreement with the Centers for Medicare and Medicaid Services does not allow us to share the data.
